# Lutetium-177 DOTATATE for the Treatment of Esthesioneuroblastoma: A Case Report

**DOI:** 10.1155/crom/5563379

**Published:** 2025-11-06

**Authors:** Vaneza Avila-Rodriguez, Nicolle Wagner-Gutiérrez, Javier Jacobo, Diego Pineda, Alejandro Gonzalez, Jorge Aristizabal, Ivan Bobadilla, Natalia Sánchez, Jairo A. Zuluaga, Andrés F. Cardona

**Affiliations:** ^1^Neuro-Oncology Unit, Luis Carlos Sarmiento Angulo Cancer Treatment and Research Center (CTIC), Bogotá, Colombia; ^2^GIGA/TERA Research Groups, CTIC/Universidad El Bosque, Bogotá, Colombia; ^3^University of North Carolina at Chapel Hill, School of Global Public Health, Chapel Hill, North Carolina, USA; ^4^Radiotherapy Oncology Unit, Luis Carlos Sarmiento Angulo Cancer Treatment and Research Center (CTIC), Bogotá, Colombia; ^5^Foundation for Clinical and Applied Cancer Research-FICMAC, Bogotá, Colombia

**Keywords:** esthesioneuroblastoma, lutetium-177 DOTATATE, peptide receptor radionuclide therapy

## Abstract

Esthesioneuroblastoma (ENB), also known as olfactory neuroblastoma, is a rare neuroendocrine malignancy arising from the olfactory neuroepithelium. It exhibits a wide range of biological behaviors, from indolent to highly aggressive disease, often requiring a multimodal treatment approach involving surgical resection, radiotherapy, and chemotherapy, though outcomes remain variable. The overexpression of somatostatin receptors (SSTRs), particularly SSTR2, has led to the exploration of peptide receptor radionuclide therapy (PRRT) with lutetium-177 DOTATATE (Lu-177) as a targeted option in refractory cases. We present the case of a 64-year-old woman with recurrent, metastatic ENB, Kadish Stage D (T2N0M0G2). After undergoing surgical resection, intensity-modulated radiotherapy (IMRT), and systemic chemotherapy (cisplatin/etoposide), the patient experienced disease progression, prompting the initiation of targeted therapy with sunitinib. Given high SSTR expression detected on Gallium-68 PET imaging, four cycles of PRRT with Lu-177 DOTATATE were introduced with adverse effects (Grade 1 fatigue, nausea, leukopenia, anemia, and thrombocytopenia). While initial tumor regression was observed, subsequent progression necessitated further stereotactic body radiotherapy (SBRT) and temozolomide. This case highlights the therapeutic potential of PRRT with Lu-177 DOTATATE in treating refractory SSTR-expressing ENB. A multidisciplinary approach that integrates surgery, radiotherapy, systemic therapy, and theragnostic strategies remains essential to optimizing patient outcomes.

## 1. Introduction

Esthesioneuroblastoma (ENB), also known as olfactory neuroblastoma, is a rare malignancy arising from the olfactory neuroepithelium in the upper nasal cavity. First described by Berger and Luc in 1924, ENB accounts for approximately 3%–6% of nasal cavity neoplasms and 0.3% of upper digestive tract malignancies, with an estimated incidence of 0.4 per million. Fewer than 1200 cases have been reported to date [1].

ENB demonstrates a broad spectrum of clinical behavior, ranging from indolent to highly aggressive. Computed tomography (CT) and magnetic resonance imaging (MRI) are key to its initial diagnosis and staging [2]. Histologically, ENB is characterized by small, round, blue cells with hyperchromatic nuclei, small nucleoli, a high nucleus-to-cytoplasm ratio, and, in high-grade tumors, pleomorphism and increased mitotic activity. These tumors express neuron-specific enolase (NSE), synaptophysin, chromogranin, CD56, and somatostatin receptors (SSTRs), particularly somatostatin receptor Type 2 (SSTR2) [3].

The standard of care for localized ENB consists of surgical resection with negative margins, followed by adjuvant radiotherapy, typically using intensity-modulated radiotherapy (IMRT) or proton beam therapy, to optimize local disease control. Despite aggressive locoregional management, ENB has a high recurrence rate, with up to 40%–60% of patients relapsing within 5 years, often presenting with cervical lymph nodes or distant metastases. While systemic chemotherapy remains a cornerstone for treating recurrent or metastatic disease, therapeutic responses vary significantly. Cisplatin and etoposide-based regimens have demonstrated transient efficacy, though long-term disease control remains elusive [4]. Given the neuroendocrine characteristics of ENB, the presence of SSTRs, particularly SSTR2, has led to the exploration of PRRT as a novel therapeutic strategy; PRRT using Lutetium-177 DOTATATE (Lu-177 DOTATATE) has emerged as a promising targeted therapy for SSTR-expressing tumors [5].

Despite advances in multimodal treatment approaches, including surgical resection, radiotherapy, and systemic therapy, recurrent and metastatic ENB remain a therapeutic challenge. The overexpression of SSTRs in ENB has led to the exploration of PRRT with Lu-177 DOTATATE as a targeted treatment option in refractory cases. This report highlights the role of PRRT in the management of ENB and discusses its therapeutic potential as part of a comprehensive, multidisciplinary treatment strategy.

## 2. Case Report

A 64-year-old woman presented with intermittent episodes of epistaxis. Given her symptoms, an axial CT scan of the paranasal sinuses revealed a tumor localized to the cribriform plate of the ethmoid bone. The patient subsequently underwent skull base resection. Pathological examination identified a small, round, blue cell tumor. Immunohistochemistry (IHC) was positive for NSE and chromogranin, while S100 and AE1/3 markers were negative. The tumor was classified as Hyams Grade 2, consistent with the neuroblastoma subtype of ENB.

Three years postoperatively, the patient experienced a recurrent episode of epistaxis. Brain MRI revealed an intermediate-intensity mass measuring 27 × 19 mm, extending into the endocranial cavity and causing bilateral frontal compression. A second skull tumor resection confirmed histopathological findings similar to the initial diagnosis. However, residual disease was detected in the anterior cranial fossa, though no pathological findings were noted in the vertebral column. To optimize disease control, complete resection of the residual tumor was performed, followed by IMRT. Follow-up imaging showed no evidence of residual disease or recurrence.

The patient remained relapse-free for 4 years. During routine follow-up, imaging identified suspected cervical lymphadenopathy and interaortocaval abdominal lymph node involvement, subsequently confirmed by positron emission tomography–computed tomography (PET-CT). A left cervical lymphadenectomy was performed, and histopathological analysis revealed one out of five lymph nodes positive for ENB, with IHC expression of CK, CD99, p16, and p63 in neck Levels I, II, and III. The patient underwent stereotactic body radiotherapy (SBRT) to the skull base and retroperitoneal lymph nodes, receiving a total dose of 2500 cGy, delivered at 500 cGy per fraction. This resulted in radiological evidence of disease regression.

**Figure 1:**
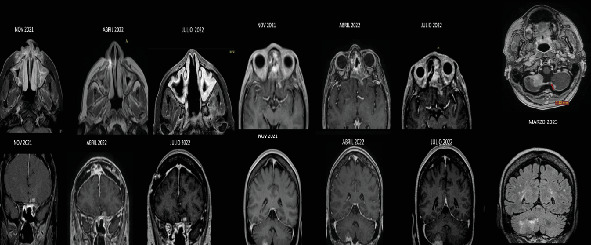
November 2021 (top row, left): Baseline study shows the enhancing lesion centered in the left paraclinoid region. April 2022 (top row, middle): Interval progression of the residual lesion in the left paraclinoid region is noted. Additionally, new extra-axial enhancement is also noted in the frontal lobe, suggestive of progressive extension. July 2022 (top row, right): Postsurgical imaging confirms the surgical intervention on the frontal lobe component, while further progression is observed in the left paraclinoid lesion along with the emergence of a new enhancing focus in the right cerebellum. May 2023 (bottom row): The latest images reveal definitive progression of the right cerebellar lesion, confirming ongoing disease advancement.

One year later, the patient developed left-sided ptosis. Sequential imaging revealed a new lesion, prompting repeat SBRT. Given the tumor's aggressive behavior and evolving characteristics, systemic chemotherapy with cisplatin and etoposide was initiated. The patient tolerated treatment well, and response evaluation after six cycles showed a partial response (PR), with normalization of chromogranin, homovanillic acid, and vanillylmandelic acid levels. However, global metabolic activity and tumor burden continued to increase. NGS identified a mitotic index of 2/10 (CGA), VHL gene loss, low TMB, and MSI-low status. Based on these findings, treatment with sunitinib was initiated, achieving disease control until resection of a lesion in the nasopharynx and posterior nasal septum (Figure 1).

**Figure 2:**
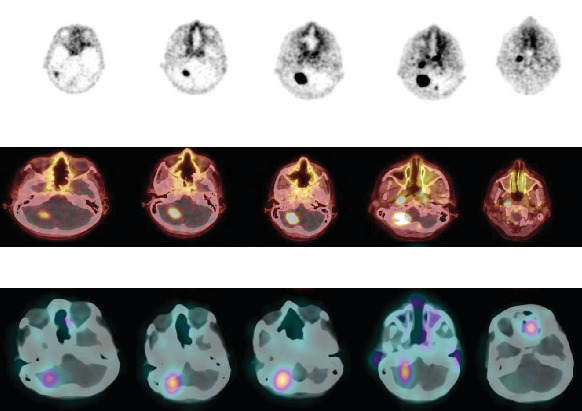
Multimodal imaging of a brainstem lesion following administration of a ^177^Lu-labeled theranostic agent. (a) Serial planar whole-brain scintigraphy at multiple time points postinjection demonstrates evolving radiotracer biodistribution, with progressive focal accumulation in the dorsal brainstem region. (b) Coregistered SPECT/CT axial slices provide anatomical localization of tracer uptake, highlighting discrete radiotracer concentration within the pontine area, with surrounding physiologic distribution in cranial structures. The integration of functional and anatomical data enables precise lesion characterization. (c) Volumetric 3D fusion images combining SPECT and MRI datasets illustrate the spatial distribution of ^177^Lu uptake relative to normal brain anatomy, facilitating assessment of metabolic activity within the tumor bed. Areas of high-intensity signal correspond to regions of active disease with preserved blood–brain barrier permeability.

**Figure 3:**
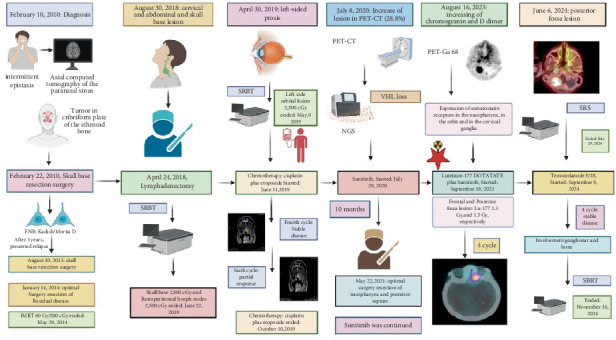
Chronological overview of the clinical course, treatments, and outcomes in a patient with advanced esthesioneuroblastoma. ENB, esthesioneuroblastoma; SRBT, stereotactic body radiotherapy; IMRT, intensity-modulated radiotherapy; PET-CT, positron emission tomography–computed tomography; PET-Ga68, Gallium-68 PET imaging; VHL, von Hippel–Lindau gene; NGS, next-generation sequencing; Lu-177 DOTATATE, lutetium-177 DOTATATE; SRS, stereotactic radiosurgery; SBRT, stereotactic body radiation therapy. The timeline illustrates key diagnostic events, surgical interventions, systemic therapies (including cisplatin–etoposide, sunitinib, Lu-177 DOTATATE, and temozolomide), and corresponding responses as determined by imaging and clinical assessments.

Despite prior interventions, disease progression was confirmed on Gallium-68 PET imaging, which showed high SSTR expression in the orbit, longitudinal paranasal sinus, nasopharynx, and cervical lymph nodes. The case was reviewed in a multidisciplinary tumor board, which recommended combination therapy with Lu-177 DOTATATE and the TKI sunitinib due to high SSTR expression on 68Ga-PET imaging and genomic evidence of *VHL* gene loss, which is known to confer sensitivity to antiangiogenic agents such as sunitinib. The patient received Lu-177 DOTATATE therapy (four cycles, each administered at a dose of 7.4 GBq [200 mCi]) every 8 weeks by intravenous administration, in accordance with standard PRRT protocols, which was well tolerated, with only Grade 1 adverse events, including fatigue, mild nausea, leukopenia, anemia, and thrombocytopenia during Cycle 2, that were transient and did not require intervention. Renal function was monitored before each cycle, with no evidence of clinically significant nephrotoxicity. A sustained clinical and radiographic response was achieved, with disease control lasting approximately 12 months following treatment initiation (Figure 2). Thereafter, imaging revealed disease progression characterized by necrosis, peritumoral edema, and new lymph node involvement in the cervical region and vertebral column. These findings were subsequently managed with SBRT and systemic therapy with temozolomide (Figure 3).

## 3. Discussion

The ENB, or olfactory neuroblastoma, is a rare malignant neoplasm originating from the olfactory neuroepithelium. The most significant prognostic factors influencing patient outcomes include Hyams grade, lymph node involvement, and Kadish stage. Management typically requires a multimodal approach incorporating surgery, radiotherapy, and systemic therapy [1]. Despite aggressive initial management, recurrence and metastasis remain significant challenges; therefore, novel therapeutic multimodal strategies, including targeted therapies and PRRT with Lu-177 DOTATATE, have emerged [2].

This case involved a patient initially classified as Kadish Stage B, who underwent surgical resection followed by IMRT, achieving temporary disease control. Tumor recurrence necessitated additional surgery and SBRT, and as the disease progressed despite systemic therapy with cisplatin–etoposide, sunitinib was introduced. Given the high SSTR expression detected on imaging, PRRT with Lu-177 DOTATATE was initiated, highlighting its potential role in treating refractory ENB. This case underscores the challenge of selecting optimal treatment sequencing in ENB, particularly in cases where conventional multimodal approaches fail to prevent disease progression.

### 3.1. Current Standard of Care and Recurrence Patterns

The current standard of care for localized ENB involves surgical resection followed by adjuvant radiotherapy, typically IMRT or proton beam therapy, to maximize locoregional disease control. A meta-analysis by Dulguerov et al. demonstrated superior survival outcomes with a combined approach, reporting 5-year survival rates of 65% for surgery plus radiotherapy, 48% for surgery alone, 47% for surgery with chemoradiation, and 37% for radiotherapy alone [6]. The SEER database analysis by Platek et al., which examined 511 ENB patients from 1973 to 2006, further reinforced the importance of a multimodal approach, with 61% of patients receiving surgery and RT. The 5-year survival rates were 73% for the surgery and RT group, 68% for surgery alone, and 35% for RT alone [7].

Despite aggressive treatment, recurrence rates remain high, with distant metastases developing in up to 40% of cases [5]. In this case, the patient experienced relapses at the skull base, cervical lymph nodes, and abdominal lymph nodes. Although lymph node metastases are uncommon at initial presentation, up to 27% of ENB patients eventually develop nodal disease. A meta-analysis by Gore and Zanation reviewing 33 studies found that 20.2% of patients, 137 of 678, developed neck metastases. The presence of nodal disease significantly worsened prognosis, increasing recurrence rates and reducing survival. The study also emphasized surgical neck dissection combined with adjuvant radiotherapy (IMRT or proton therapy) as the most effective strategy for local disease control [8].

### 3.2. Systemic Therapy and the Role of PRRT

Systemic therapy plays a critical role in treating recurrent and metastatic ENB. In this case, after developing left-sided ptosis, the patient was treated with cisplatin and etoposide, a regimen with transient efficacy in neuroendocrine tumors but limited long-term disease control. A retrospective case study of 10 patients with ENB high-grade Kadish C by McElroy et al. showed limited response to platinum-based chemotherapy, with tumor regression in 50% of cases (mean duration: 9.3 months) and significantly lower survival (32.2 vs. 139.5 months for low-grade tumors). Cisplatin–etoposide remains crucial in high-grade and metastatic cases, with optimal efficacy in multimodal strategies [9]. Subsequently, a new disease progression was documented, NGS, which identified VHL gene loss, a mutation that is not usually found in ENB, but a feature linked to increased sensitivity to antiangiogenic therapies such as sunitinib, which was then initiated [10, 11].

Given its neuroendocrine differentiation, 82% of ENB expresses SSTRs, particularly SSTR2, which targets PRRT. In this case, high SSTR expression guided the decision to initiate Lu-177 DOTATATE therapy, which initially led to tumor regression [12]. The benefit of Lu-177 DOTATATE as therapy in neuroendocrine tumors was demonstrated in the NETTER-1 clinical trial [13].

### 3.3. Evidence Supporting PRRT in ENB

Few studies have evaluated PRRT in ENB, but available data suggest promising outcomes. In a study by Zhi et al., five patients with Kadish Stage D ENB underwent SSTR-directed theragnostic imaging using ^68^Ga-DOTATOC PET-CT, which identified SSTR-positive lesions in 100% of cases. Patients received a median of four cycles (range: 2–6) of PRRT with a median administered dose of 7.7 GBq per cycle. The overall treatment response demonstrated a PR in 60% of patients, stable disease in 20%, and progressive disease in 20%. The median progression-free survival (PFS) was 29 weeks (range: 13–53 weeks) [14].

Another study by Hasan et al. evaluated seven patients with recurrent, unresectable, and metastatic ENB treated with Lu-177 DOTATATE, achieving disease control in 86% of cases, with a median PFS of 17 months and overall survival of 32 months. PRs were observed in 57% of patients, while 29% had stable disease [15]. The LUTHEE trial (NCT03454763) is currently ongoing and actively enrolling patients; it is a Phase II randomized clinical trial that aims to compare the efficacy and safety of two different administration schedules of Lu-177 DOTATATE PRRT in patients with SSTR2-positive tumors, including neuroendocrine tumors and potentially ENB [16].

### 3.4. Limitations and Future Directions

Although PRRT has demonstrated efficacy, its role within the broader treatment paradigm for ENB remains uncertain. Currently, it is primarily used as salvage therapy in refractory cases, but emerging evidence suggests that earlier integration into treatment algorithms may improve patient outcomes. Additionally, combination strategies involving TKIs, immune checkpoint inhibitors, or other systemic therapies could offer synergistic effects, warranting further investigation.

A significant limitation in ENB treatment is the rarity of the disease, which restricts the availability of randomized clinical trials directly comparing therapeutic efficacy. Most available data are derived from retrospective studies, case reports, and small institutional series, resulting in a lack of standardized treatment algorithms and evidence-based therapeutic strategies. Furthermore, the absence of validated predictive biomarkers beyond SSTR imaging may limit the ability to accurately assess treatment response. Identifying molecular markers associated with PRRT efficacy could help refine patient selection criteria and optimize therapeutic decision-making.

Despite its potential, PRRT alone may not provide long-term disease control in all cases. In this case, the patient developed recurrent disease in the orbit, nasopharynx, and cervical lymph nodes, necessitating SBRT with temozolomide, an alkylating agent frequently used in neuroendocrine tumors. These findings further underscore the importance of an individualized, multimodal treatment approach, integrating theragnostic strategies with surgery, radiotherapy, and systemic therapy to optimize disease control.

## 4. Conclusion

This case highlights the therapeutic complexity of ENB and illustrates the clinical value of an individualized, multidisciplinary treatment approach. To our knowledge, it is among the few documented reports combining PRRT with Lu-177 DOTATATE and sunitinib in a patient with refractory, SSTR2-positive ENB, guided by both molecular profiling and theragnostic imaging. This integration of targeted systemic therapy with radionuclide treatment offers a novel therapeutic approach in a rare disease. The case contributes valuable insight into sequencing strategies and supports further investigation into the role of PRRT in ENB management.

## Data Availability

Data sharing is not applicable to this article as no datasets were generated or analyzed during the current study.

## References

[1] Bak M., Wein R. O. (2012). Esthesioneuroblastoma. *Hematology/Oncology Clinics of North America*.

[2] Kumar R. (2015). Esthesioneuroblastoma: Multimodal Management and Review of Literature. *World Journal of Clinical Cases*.

[3] Ni G., Pinheiro-Neto C. D., Iyoha E. (2023). Recurrent Esthesioneuroblastoma: Long-Term Outcomes of Salvage Therapy. *Cancers (Basel)*.

[4] Aljumaily R. M., Nystrom J. S., Wein R. O. (2011). Neoadjuvant Chemotherapy in the Setting of Locally Advanced Olfactory Neuroblastoma With Intracranial Extension. *Rare Tumors*.

[5] Cracolici V., Wang E. W., Gardner P. A. (2021). SSTR2 Expression in Olfactory Neuroblastoma: Clinical and Therapeutic Implications. *Head and Neck Pathology*.

[6] Dulguerov P., Allal A. S., Calcaterra T. C. (2001). Esthesioneuroblastoma: A Meta-Analysis and Review. *Lancet Oncology*.

[7] Platek M. E., Merzianu M., Mashtare T. L. (2011). Improved Survival Following Surgery and Radiation Therapy for Olfactory Neuroblastoma: Analysis of the SEER Database. *Radiation Oncology*.

[8] Gore M. R., Zanation A. M. (2009). Salvage Treatment of Late Neck Metastasis in Esthesioneuroblastoma: A Meta-Analysis. *Archives of Otolaryngology – Head & Neck Surgery*.

[9] McElroy E. A., Buckner J. C., Lewis J. E. (1998). Chemotherapy for Advanced Esthesioneuroblastoma: The Mayo Clinic Experience. *Neurosurgery*.

[10] Gay L. M., Kim S., Fedorchak K. (2017). Comprehensive Genomic Profiling of Esthesioneuroblastoma Reveals Additional Treatment Options. *Oncologist*.

[11] Preusser M., Hutterer M., Sohm M. (2010). Disease Stabilization of Progressive Olfactory Neuroblastoma (Esthesioneuroblastoma) Under Treatment With Sunitinib Mesylate. *Journal of Neuro-Oncology*.

[12] Lechner M., Takahashi Y., Turri-Zanoni M. (2022). Clinical Outcomes, Kadish-INSICA Staging and Therapeutic Targeting of Somatostatin Receptor 2 in Olfactory Neuroblastoma. *European Journal of Cancer*.

[13] Strosberg J., El-Haddad G., Wolin E. (2017). Phase 3 Trial of ^177^Lu-Dotatate for Midgut Neuroendocrine Tumors. *New England Journal of Medicine*.

[14] Zhi Y., Serfling S. E., Groener D. (2025). Somatostatin Receptor-Directed Theranostics in Esthesioneuroblastoma. *Clinical Nuclear Medicine*.

[15] Hasan O. K., Ravi Kumar A. S., Kong G. (2020). Efficacy of Peptide Receptor Radionuclide Therapy for Esthesioneuroblastoma. *Journal of Nuclear Medicine*.

[16] NCT03454763.

